# IL-6, TNF-α, and IL-10 levels/polymorphisms and their association with type 2 diabetes mellitus and obesity in Brazilian individuals

**DOI:** 10.1590/2359-3997000000254

**Published:** 2017-02-01

**Authors:** Kathryna Fontana Rodrigues, Nathalia Teixeira Pietrani, Adriana Aparecida Bosco, Fernanda Magalhães Freire Campos, Valéria Cristina Sandrim, Karina Braga Gomes

**Affiliations:** 1 Instituto de Ciências Biológicas Universidade Federal de Minas Gerais Belo Horizonte MG Brasil Instituto de Ciências Biológicas, Universidade Federal de Minas Gerais (UFMG), Belo Horizonte, MG, Brasil; 2 Instituto de Ensino e Pesquisa Santa Casa de Belo Horizonte Belo Horizonte MG Brasil Instituto de Ensino e Pesquisa, Santa Casa de Belo Horizonte, Belo Horizonte, MG, Brasil; 3 Faculdade de Farmácia UFMG Belo Horizonte MG Brasil Faculdade de Farmácia, UFMG, Belo Horizonte, MG, Brasil; 4 Instituto de Biociências Universidade Estadual Paulista Júlio de Mesquita Filho Botucatu SP Brasil Instituto de Biociências, Universidade Estadual Paulista Júlio de Mesquita Filho (Unesp), Botucatu, SP, Brasil

**Keywords:** Type 2 diabetes mellitus, polymorphisms, interleukin-6, interleukin-10, tumor necrosis factor-alpha

## Abstract

**Objective:**

This study aimed to investigate the association of plasma TNF-α, IL-6, and lL-10 levels and cytokine gene polymorphisms [TNF-α (-308 G→A), IL-6 (-174 C→G) and IL-10 (-1082 A→G, -819 T→C and -592 A→C)] in type 2 diabetes mellitus (T2DM) and obese patients.

**Subjects and methods:**

One hundred and two T2DM patients and 62 controls were included in this study. Cytokine plasma levels were measured by the Cytometric Bead Array method. Genotyping was carried out by the polymerase chain reaction.

**Results:**

IL-6 levels were significantly different between T2DM patients and controls. Interestingly, IL-6 levels were higher in T2DM patients with BMI > 30 kg/m^2^ compared with other patients and obese controls. The genotype and allele frequencies were similar between patients and controls. In the T2DM group, the SNP IL-10 -819 T/C showed a difference between the cytokine level and genotypes: IL-10 level in the TT genotype was significantly higher when compared to CC genotype.

**Conclusions:**

These results suggest an association between IL-6 levels and obesity, and IL-10 levels and the SNP -819 T/C in T2DM. Knowledge of these variants in T2DM might contribute to a better understanding of the role of inflammation in the etiology and progression of this disease.

## INTRODUCTION

Type 2 diabetes mellitus (T2DM) is a heterogeneous group of metabolic disorders characterized by chronic hyperglycemia and represents a significant global health problem (1). According to the International Diabetes Federation (IDF), diabetes mellitus is a major metabolic disease affecting approximately 415 million people worldwide and it is expected to reach 642 million in 2040 (2).

The pathogenesis of insulin resistance and T2DM has been associated with a subclinical chronic inflammation and activation of the immune system; however, what triggers this inflammation is still unclear (3,4). Some studies have shown that T2DM patients have higher levels of inflammatory markers such as interleukin-6 (IL-6), C reactive protein (CRP), plasminogen activator inhibitor-1 (PAI-1), tumor necrosis factor-α (TNF-α), vascular cell adhesion molecule-1 (VCAM-1), and intercellular adhesion molecule-1 (ICAM-1) (5-10).

Furthermore, it is known that obesity, especially the visceral type, is an independent risk factor for T2DM development (11). In fact, adipose tissue is an endocrine organ that co-regulates whole-body metabolism. It is able to produce a variety of cytokines (TNF-α, IL-6, IL-1β) and other bioactive products, such as leptin, resistin, and monocyte chemoattractant protein-1 (MCP-1/CCL2) (12,13). Adipose tissue in an obese individual is characterized by the presence of pro-inflammatory immune cells (CD8^+^ T lymphocytes, IFN-γ^+^ Th1 cells, B cells, mast cells, neutrophils, and M1 macrophages) attracted by chemokines secreted from stressed adipocytes in response to lipid overload (14).

The expression of pro- and anti-inflammatory cytokines may be modulated by single nucleotide polymorphisms (SNPs) located in the regulatory regions of genes (15,16). Some studies have investigated the association among TNF-α, IL-6, and IL-10 gene polymorphisms with metabolic diseases (17-25). Despite these reports examining the association of inflammation markers and SNPs in cytokine genes, much controversy remains as to their role in diabetes occurrence (26-34).

In this study, we evaluated the role of cytokines in T2DM and obesity by measuring plasma TNF-α, IL-6, and IL-10 levels. We also investigated whether these levels are modulated by polymorphisms located in the regulatory regions of genes (TNF-α (-308 G/A, rs1800629), IL-6 (-174 C/G, rs1800795), and IL-10 (-1082 G/A, rs1800896; -819 T/C, rs1800871; and -592 A/C, rs1800872)). Higher IL-6 levels were found in T2DM patients and our results suggest that obesity acts synergistically with T2DM by modulating the increase of this cytokine. Although this study failed to demonstrate that these polymorphisms could modulate TNF-α, IL-6, and IL-10 plasma levels, the IL-10 -819 T/C polymorphism seems to influence IL-10 levels in T2DM.

## SUBJECTS AND METHODS

### Ethical aspects

This study was approved by the Ethics Committee of the Federal University of Minas Gerais (Minas Gerais, Brazil)-ETIC 0062.0.203.000-11-and Santa Casa Hospital (Minas Gerais, Brazil)-059/2011; in accordance with the Helsinki Declaration. Informed consent was obtained from all subjects.

### Study design

This cross-sectional study was conducted with 102 patients with clinical and laboratory diagnosis of T2DM (19 men and 83 women) and 62 non-diabetic controls (12 men and 50 women); both groups were aged from 32 to 70 years and matched by gender, age, and body mass index (BMI) in a 2:1 case/control proportion, according to the sample calculation based on the mean values for each cytokines level obtained from a small sample of the groups (power = 0.95; significance level = 0.05). The patients were selected from the Clinic of Endocrinology (Santa Casa Hospital, Minas Gerais, Brazil), and the controls were selected from the local community between June 2012 and September 2013. T2DM diagnosis was based on the American Diabetes Association (ADA) criteria (1). The controls showed normal levels of fasting glucose (60-99 mg/dL) and no use of hypoglycemic drugs. Were excluded subjects older than 70 years, pregnant, with cancer, autoimmune disease, recent history of cardiovascular disease (heart attack, stroke, thrombosis in the last five years), and current or recent infections and/or inflammatory processes.

### Clinical and laboratorial data

Clinical (gender, age, BMI, waist circumference, waist-hip ratio, T2DM onset, and hypertension), and laboratorial data (fasting glucose, HbA1c, and post-prandial glucose) were obtained for all of the T2DM patients through interviews and medical records. The criteria used for determining hypertension were: systolic blood pressure ≥ 140 mmHg or diastolic blood pressure ≥ 80 mmHg, or use of antihypertensive drugs, and comply with the criteria adopted by the ADA (1). Clinical data (gender, age, BMI, waist circumference, and waist-hip ratio) for the controls were obtained through interviews.

The fasting glucose in the control group was measured in serum samples after eight hours fasting. The serum samples were centrifuged at 1,100 x g for 20 min at 25ºC and the assays performed immediately. The tests were performed using the enzyme-colorimetric method, BTR 811 spectrophotometer (Biotron, Minas Gerais, Brazil), and Glicose-PP kit (Gold Analisa, Minas Gerais, Brazil), following the manufacturer’s instructions. The concentrations of fasting glucose were expressed in mg/dL.

Serum samples were used for quantification high sensitivity C reactive protein (hs-CRP). They were centrifuged at 1,100 x g for 20 min at 25ºC and stored at -80ºC until analysis. The tests were performed using the immunoturbidimetric method, System Vitros Chemistry 5.1 FS (Ortho Clinical Diagnostics, New York, USA), and hsCRP VITROS Chemistry Products (Ortho Clinical Diagnostics, New York, USA), following the manufacturer’s instructions. All samples were assayed at the same time. The concentrations of hs-CRP were expressed in mg/L.

### Determination of cytokine plasma levels

Samples collected in EDTA were centrifuged at 1,100 x g for 20 min at 25ºC to obtain plasma, which was stored at -80ºC until analysis. Data acquisition and analysis were performed in an LSR Fortessa^TM^ flow cytometer (BD Biosciences Pharmingen, California, USA) using FCAP Array Software version 1.0.1. TNF-α, IL-6, and IL-10 levels were determined by the Cytometric Bead Array (CBA) method using Human Enhanced Sensitivity Flex Set Systems (BD Biosciences Pharmingen, California, USA), following the manufacturer’s instructions. All samples were assayed at the same time. The concentrations of each cytokine were expressed in fg/mL.

### Cytokine gene polymorphism analysis

Genomic DNA was extracted from whole blood collected in EDTA using Biopur Mini Spin Kit (Biometrix, São Paulo, Brazil). The polymorphisms were determined using the Cytokine Genotyping Tray Kit (One Lambda Inc., California, USA), which employs Polymerase Chain Reaction-Sequence Specific Primers (PCR-SSP), followed by electrophoresis in 2.5% agarose gel stained with GelGreen Stain (Biotium Inc., California, USA). In order to evaluate the reproducibility rate of genotyping, 10% of the samples in both groups were randomly selected to be re-genotyped. The results showed 100% agreement. The polymorphisms analyzed in the present study were: TNF-α (-308 G/A, rs1800629), IL-6 (-174 C/G, rs1800795), IL-10 (-1082 G/A, rs1800896; -819 T/C, rs1800871; and -592 A/C, rs1800872).

### Statistical analysis

Deviations from Hardy-Weinberg equilibrium (HWE) were tested using an exact test (available at: http://genepop.curtin.edu.au/genepop_op1.html). All of the statistical analyses were performed with Statistical Package of the Social Sciences (SPSS) version 17.0. An analysis of normality was performed by Shapiro-Wilk test. Data are presented as “mean ± (standard deviation-SD)” (parametric variables), “median (interquartile range-IQR)” (non-parametric variables), or “percentage of total (categorical variables)”.

Comparisons between the two groups were made with Student’s *t*-test for parametric variables and the Mann-Whitney test for non-parametric variables. Comparisons of non-parametric variables between three groups were performed with the Kruskal-Wallis test. When differences were detected, they were compared in pairs by the Mann-Whitney method, followed by Bonferroni’s Correction. The comparison of categorical variables was performed using the chi-square test (χ^2^).

Differences in genotype and allele frequencies between the groups (T2DM patient and control) were tested by Pearson’s χ^2^-test or Fisher’s Test. IL-10 haplotype estimation was conducted by PHASE software version 2.1. We excluded haplotypes whose frequencies were less than 5%. The differences in the haplotype frequencies between the groups were tested by χ^2^-test.

Linear regression analysis was performed for evaluating the confounding influence of variables in cytokines plasma levels. Gender, age, BMI, waist circumference, waist-hip ratio, and fasting glucose were considered as independent variables.

Spearman’s correlations were computed only in the T2DM patient group to assess correlations between cytokine levels, anthropometric, and laboratorial data. A *p*-value < 0.05 was considered statistically significant.

## RESULTS

Clinical and laboratorial characteristics of T2DM patients and controls are summarized in Table 1. Groups were matched by gender, age, and BMI (p > 0.05 for all). Waist circumference and waist-hip ratio were higher in T2DM patients than in controls (p < 0.0001 for both). When analyzing cytokine levels in two groups, IL-6 levels were higher in the diabetic patients (p = 0.001), and no significant differences were observed for TNF-α (p = 0.332) and IL-10 (p = 0.317). Diabetic patients on treatment with insulin (n = 18, 17.65%), oral antidiabetic drugs (n = 17, 16.67%) or insulin plus oral antidiabetic drugs (n = 67, 65.68%) show similar levels of cytokines compared with each other (p > 0.05 for all-Supplementary Material-SM1).

**Table 1:** Clinical and laboratorial characteristics in T2DM patients and controls

Parameters	T2DM (n = 102)	Control (n = 62)	p
Gender (Male/Female) %	18.6 / 81.4	19.3 / 81.7	0.908
Age (years)	56 (12)	53 (18)	0.358
BMI (kg/m^2^)			
BMI < 25	22.6 (3.7)	23.4 (2.8)	0.503
25 ≤ BMI < 30	28.3 (3.0)	27.6 (1.9)	0.215
BMI ≥ 30	37.8 (9.2)	39.3 (11.0)	0.850
Waist circumference (cm)	108.1 ± 16.8	96.9 ± 16.2	< 0.0001*
Waist-hip ratio	1.0 ± 0.1	0.9 ± 0.1	< 0.0001*
T2DM onset (%)^a^			
≤ 10 years	40.2	Not applicable	-
> 10 years	56.9		-
Hypertension (Yes/No) %	92.2 / 7.8	-	-
Fasting glucose (mg/dL)	126.5 (79.0)	85.3 (10.3)	< 0.0001*
Post-prandial glucose (mg/dL)	203.0 (118.0)	-	-
HbA1c (%)	8.9 ± 1.9	-	-
hs-CRP (mg/L)	3.5 (6.6)	2.7 (2.6)	0.094
TNF-α (fg/mL)	91 (42)	95 (64)	0.332
IL-6 (fg/mL)	805 (993)	476 (516)	0.001*
IL-10 (fg/mL)	194 (67)	185 (57)	0.317

BMI: body mass index; HbA1c: glycated hemoglobin; hs-CRP: high sensitivity C reactive protein; TNF-α: tumor necrosis factor-alpha; IL-6: interleukin-6; IL-10: interleukin-10.

Normal variables (waist circumference, waist-hip ratio, and HbA1c): the data are shown as “mean ± SD”. No normal variables (age, BMI, fasting glucose, post-prandial glucose, hs-CRP, TNF-α, IL-6, and IL-10): the data are shown as “median (IQR)”. Categorical variables (gender, T2DM onset, and hypertension): the data are shown as “percentage of total”.

^a^ Missing data for three patients.

* p < 0.05 was considered statistically significant.

We have evaluated cytokines levels according to BMI categories (BMI < 25 – lean, 25 ≤ BMI < 30 – overweight, and BMI ≥ 30 kg/m^2^ – obese) within each group (T2DM patient or control) and between these groups (Table 2). In T2DM group, only IL-6 levels were different between patients (p = 0.001) when compared to those with BMI < 25 kg/m^2^ versus BMI ≥ 30 kg/m^2^ (p = 0.001). Comparison among 25 ≤ BMI < 30 kg/m^2^ versus BMI ≥ 30 kg/m^2^ was not significant after Bonferroni’s correction (p > 0.02). No differences were found in the cytokine levels considering only control group. Finally, when comparing T2DM patients versus controls, IL-6 levels were higher in obese patients than in obese controls (p = 0.019). No other associations were found for TNF-α and IL-10 levels and BMI categories.

**Table 2:** Cytokines levels in T2DM patients and controls according to BMI categories

Cytokine (fg/mL)	BMI (kg/m^2^)		T2DM (n = 102)	Control (n = 62)	p’
TNF-α	BMI < 25		86 (48)	90 (75)	0.786
	25 ≤ BMI < 30		92 (48)	86 (68)	0.748
	BMI ≥ 30		92 (42)	112 (58)	0.061
		p	0.922	0.294	
IL-6	BMI < 25		452 (412)	412 (492)	0.422
	25 ≤ BMI < 30		731 (917)	532 (439)	0.458
	BMI ≥ 30		1118 (1506)	663 (534)	0.019*
		p	0.001*	0.356	
IL-10	BMI < 25		232 (102)	192 (48)	0.172
	25 ≤ BMI < 30		188 (71)	169 (64)	0.947
	BMI ≥ 30		185 (55)	190 (37)	0.473
		p	0.085	0.353	

BMI: body mass index; TNF-α: tumor necrosis factor-alpha; IL-6: interleukin-6; IL-10: interleukin-10.

Cytokine levels: no normal variable; the data are shown as “median (IQR)”.

* p < 0.05 was considered statistically significant (comparison within the groups: T2DM or control).

* p’ < 0.05 was considered statistically significant (comparison between the groups: T2DM versus control).

Mann-Whitney test with Bonferroni’s Correction for IL-6 levels (T2DM group):

* p^1^ – p^3^: were considered statistically significant if p < 0.02.

p^1^: [BMI < 25 (T2DM) versus 25 ≤ BMI < 30 (T2DM)] = 0.334.

p^2^: [BMI < 25 (T2DM) versus BMI ≥ 30 (T2DM)] = 0.001*.

p^3^: [25 ≤ BMI < 30 (T2DM) versus BMI ≥ 30 (T2DM)] = 0.024.

We performed an analysis of genotype and allele frequencies of polymorphisms for cytokine genes TNF-α, IL-6, and IL-10. All polymorphisms were under Hardy-Weinberg equilibrium (p > 0.025). We found no differences between genotype and allele frequencies when compared T2DM and control groups (p > 0.05 for all; Supplementary Material-SM2). Additionally, no difference regarding the IL-10 haplotype analyses was observed between these groups (data not shown).

Aiming to evaluate whether polymorphisms in TNF-α, IL-6, and IL-10 genes modulate cytokine plasma levels, we compared their levels and genotypes considering all the individuals (T2DM patients and controls) and performed an analysis to predict their inheritance pattern. However, no differences were found (p > 0.05 for all; Table 3). Interestingly, when these comparisons were applied only in the T2DM patient group (Table 4), IL-10 -819 T/C polymorphism showed a difference between cytokine levels and genotypes (p = 0.021): IL-10 levels in the TT genotype (256 (207) fg/mL] were significantly higher than in the CC genotype (182 (57) fg/mL] (p = 0.006). No differences were found for IL-6 levels and -174 C/G polymorphism after Bonferroni’s correction (p > 0.02) and TNF-α levels and -308 G/A polymorphism (p > 0.05). Regarding the inheritance pattern, only IL-10 -592 A/C polymorphism showed significant differences in IL-10 levels (p = 0.039) when compared to the CC genotype (182 (57) fg/mL] and AC+AA genotypes (192 (115) fg/mL], suggesting a recessive model.

**Table 3:** Cytokines levels according to genotypes considering all the individuals (T2DM patients and controls)

Polymorphism		Cytokine (fg/mL)	p
TNF-α (-308 G/A) (rs1800629)	GG (n = 125)	95 (55)	0.705
	GA (n = 38)	90 (63)	
	AA (n = 1)	47.5^†^	
IL-6 (-174 C/G) (rs1800795)	CC (n =10)	1381 (2892)	0.361
	CG (n = 50)	565 (754)	
	GG (n = 104)	674 (770)	
IL-10 (-1082 G/A) (rs1800896)	GG (n = 20)	204 (55)	0.278
	GA (n = 73)	182 (63)	
	AA (n = 71)	185 (65)	
IL-10 (-819 T/C) (rs1800871)	TT (n = 20)	214 (136)	0.215
	TC (n = 69)	182 (69)	
	CC (n = 75)	193 (55)	
IL-10 (-592 A/C) (rs1800872)	AA (n = 21)	208 (135)	0.409
	AC (n = 68)	182 (68)	
	CC (n = 75)	193 (55)	

TNF-α: tumor necrosis factor-alpha; IL-6: interleukin-6; IL-10: interleukin-10.

Cytokine levels: no normal variable; the data are shown as “median (IQR)”.

p < 0.05 was considered statistically significant.

^†^ data only one individual.

**Table 4:** Cytokines levels according to genotypes in T2DM patients group

Polymorphism		Cytokine (fg/mL)	p	p’
TNF-α (-308 G/A) (rs1800629)	GG (n = 78)	95 (45)	0.137	-
	GA (n = 23)	85 (44)		
	AA (n = 1)	47.5^†^		
IL-6 (-174 C/G) (rs1800795)	CC^1^ (n = 7)	1901 (3167)	0.026*	p^a^ = 0.047
	CG^2^ (n = 26)	496 (746)		p^b^ = 0.210
	GG^3^ (n = 69)	878 (945)		p^c^ = 0.023
IL-10 (-1082 G/A) (rs1800896)	GG (n = 12)	202 (62)	0.181	-
	GA (n = 47)	178 (68)		
	AA (n = 43)	199 (87)		
IL-10 (-819 T/C) (rs1800871)	TT^1^ (n = 13)	256 (207)	0.021*	p^a^ = 0.066
	TC^2^ (n = 40)	184 (90)		p^b^ = 0.006*
	CC^3^ (n = 49)	182 (57)		p^c^ = 0.207
IL-10 (-592 A/C) (rs1800872)	AA (n = 14)	249 (212)	0.055	-
	AC (n = 39)	185 (91)		
	CC (n = 49)	182 (57)		

TNF-α: tumor necrosis factor-alpha; IL-6: interleukin-6; IL-10: interleukin-10.

Cytokine levels: no normal variable; the data are shown as “median (IQR)”.

* p < 0.05 was considered statistically significant.

* p’ < 0.02 was considered statistically significant (Mann-Whitney test with Bonferroni’s Correction).

p^a^: genotype 1 versus genotype 2.

p^b^: genotype 1 versus genotype 3.

p^c^: genotype 2 versus genotype 3.

^†^ data only one individual.

We investigated the correlation between cytokine plasma levels and anthropometric and laboratorial data in the T2D patients group. IL-6 levels showed a significant positive correlation with BMI (r = 0.314, p = 0.002), waist circumference (r = 0.318, p = 0.002), hs-CRP (r = 0.452, p < 0.0001), and IL-10 levels (r = 0.336, p = 0.001). No other significant correlation was observed between cytokines levels (TNF-α and IL-10) and these parameters. Furthermore, fasting glucose and hs-CRP showed a significant negative and positive correlations, respectively, with BMI (r = -0.365, p < 0.0001; r = 0.476, p < 0.0001) and waist circumference (r =- 0.278, p = 0.005; r = 0.442, p < 0.0001).

Finally, the linear regression analysis did not show an independent association between gender, age, BMI, waist circumference, waist-hip ratio, and fasting glucose with TNF-α, IL-6, and IL-10 levels (p > 0.05 for all).

## DISCUSSION

This study evaluated the importance of TNF-α, IL-6, and IL-10 levels and their association with gene polymorphisms in T2DM disease and the obesity.

Clinical and laboratorial characteristics of T2DM patients and controls showed that T2DM patients have higher waist circumferences and waist-hip ratios when compared to the controls. These results are consistent with the knowledge that not only obesity, but mainly the distribution of body fat (mostly upper body obesity), influence glucose metabolism and are independent risk factors for developing T2D (11).

Among the cytokines levels measured, only IL-6 levels were higher in the T2DM group. IL-6 is a multifunctional cytokine and is secreted by many types of cells, mainly T cells, macrophages, endothelial cells, smooth muscle cells, adipocytes, and hepatocytes (35). Furthermore, IL-6 regulates/stimulates production of cell adhesion molecules, chemotactic mediators, and acute phase protein, and mediates the release of other cytokines that amplify the inflammatory response (35-37). Similar to our result, other studies have shown that T2DM individuals have higher circulating IL-6 levels when compared to non-diabetic controls (38-43). The increased levels of IL-6 and other inflammatory markers (IL-1β, CRP) emerge as early predictors of T2DM, preceding its clinical onset (44).

Obesity is also associated with a state of low-grade inflammation (11,13) and elevated levels of IL-6, which has been commonly described in obese diabetic patients or only in obese individuals (38,45,46). We performed an analysis in order to investigate the influence of BMI in cytokine levels. For the control group, no differences were found. However, IL-6 levels in obese T2DM patients (BMI ≥ 30 kg/m^2^) were higher than lean (BMI < 25 kg/m^2^) and overweight (25 ≤ BMI < 30 kg/m^2^) patients (tendency), although the linear regression analysis has not showed an independent association between these parameters. Moreover, obese T2DM patients presented higher IL-6 levels when compared to obese controls. These results suggest that higher BMI values in T2DM are associated with increased IL-6 levels, but some other variable appears to act synergistically, since this association was not independent. When compared to the control group, T2DM seems to act synergistically with obesity to promote an increase in IL-6 levels.

Although no difference was found in TNF-α and IL-10 levels between the groups (T2DM patients and controls) and the BMI categories, these cytokines have been associated with T2DM. TNF-α is a pro-inflammatory cytokine produced by a variety of cell-types, mainly macrophages, lymphocytes, and adipocytes (13). Some studies found higher TNF-α levels in T2DM patients when compared with non-diabetic controls (39,40,43).

IL-10 is an anti-inflammatory cytokine that plays an important role in the regulation of the immune system leading to decreased cytokine production, reducing tissue factor expression, inhibiting matrix-degrading metalloproteinase, and promoting the phenotypic switching of lymphocytes to the Th2 phenotype (47). This cytokine is produced by T-cells, B-cells, monocytes, and macrophages, and it is estimated that 75% of the variation in IL-10 production is genetically determined (47). An important study showed that IL-10 level was lower in subjects with impaired glucose tolerance or T2DM when compared with subjects with normal glucose tolerance and showed an inverse correlation with BMI (48). Conversely, Al-Shukaili and cols. (49) found higher IL-10 levels in T2DM patients when compared with healthy controls. Taken together, it is not clear whether higher IL-10 levels confer protection against T2DM development by decreased pro-inflammatory cytokines production, or increased IL-10 levels in T2DM result in a compensatory response against the elevation of pro-inflammatory mediators, primarily TNF-α and IL-6.

No polymorphism showed a difference in allele and genotype frequencies when compared to T2DM patients and the control group. Therefore, no polymorphism in this study was associated with T2DM. However, the association of these polymorphisms with T2DM remains unclear. In 2011, Feng and cols. (26) in a meta-analysis did not find a significant association between TNF-α -308 G/A polymorphism and T2DM risk when considering Caucasian and Asian populations. In contrast, in 2014, a meta-analysis conducted by Zhao and cols. (27) indicated that TNF-α -308A allele could be a risk factor for the development for T2DM in Asian subjects. Similarly, Golshani and cols. (28) found that TNF-α -308 GA+AA genotypes are associated with higher risk for T2DM development in an Iranian population. According to Qi and cols. (29), IL-6 -174 C/G polymorphism is not associated with the risk of T2DM development; however, a recent study (23) shows a significant association between T2DM and IL-6 -174G allele. Finally, four recent meta-analyses evaluated the association between IL-10 gene polymorphisms (-1082 G/A, -819 T/C, and -592 A/C) and the risk of T2DM development. The -819 T/C and -592 A/C polymorphisms did not show an association with the disease in these studies (30-33). However, Li and cols. (31) and Hua and cols. (30) found an association between the -1082GA genotype and -1082G allele, respectively, with T2DM. Additionally, a case-control study conducted by Bai and cols. (34) found higher risk for T2DM development associated with -1082 GA+GG and -592 AC+AA genotypes. These conflicting results are probably related to the sample size and different genetic background of the populations. Larger scale genome studies are required to further evaluate these associations.

The plasma levels of IL-6 were significantly different between -174 C/G polymorphism genotypes, but this difference was not maintained after Bonferroni’s correction. The patients with IL-10 -819TT genotype showed higher IL-10 levels than patients with -819CC genotype. Previous studies (50,51) showed that, not only the -819T allele, but also the ATA haplotype (-1082A, -819T, -592A) are related to lower transcriptional activity and, consequently, lower IL-10 levels. However, no further study was carried out on diabetic patients to prove the association between transcriptional activity of IL-10 gene and IL-10 serum/plasma levels and -819 T/C polymorphism.

IL-6 levels were positively correlated with BMI, waist circumference, hs-CRP, and IL-10 levels. Similarly, hs-CRP also showed a significant positive correlation with BMI and waist circumference. Obesity, especially visceral obesity, is characterized by the increased size of adipocytes and recruitment of immune cells (mainly macrophages), which display a pro-inflammatory profile. These cells are responsible for the increased production of inflammatory mediators and acute phase proteins, such as IL-6 and CRP (12). Considering these events, it is expected that IL-6 and hs-CRP levels are positively correlated with each other and with anthropometric parameters that express weight gain (BMI) and the increase in upper body fat (waist circumference) in the T2DM group. Finally, the positive correlation between IL-6 and IL-10 levels showed that, in T2DM, the increase of pro-inflammatory mediators may cause a compensatory increase in anti-inflammatory cytokines to control subclinical inflammation.

The small sample size, owing to the strict selection criteria for patients and controls, and absence of functional analysis of polymorphisms can be considered as the main limitations of this study, since the effect of the polymorphisms on protein activity was not evaluated. Indeed, the statistical power became lower when the total number of the participants in each group was classified according to BMI categories, but as cytokine levels are associated with adiposity, this selection was necessary in order to avoid a bias in the results.

Therefore, further studies with a much larger sample exploring other populations (different genetic background) and others clinical characteristics as a practice of physical activities, are needed to better understand the role of these polymorphisms in the subclinical inflammation in T2DM.

In conclusion, few studies have evaluated inflammatory markers and cytokines genes polymorphisms in T2DM Brazilian patients. Considering the increased number of diabetic patients in Brazil and the population’s genetic background, improved knowledge on the markers that contribute to the etiology and progression of T2DM are important for prevention, diagnosis, and follow-up of this disease.

Taken together, our results show that IL-6 and IL-10 levels and the SNP -819 T/C in IL-10 gene are associated with the subclinical inflammation in the T2DM. Moreover, the association between IL-6 levels and obesity in T2DM indicates that weight control may be an action adopted for preventing inflammatory status in T2DM.

## SUPPLEMENTARY MATERIAL

**Table SM1:** Cytokines levels according to types of pharmacological treatment in T2DM group

Cytokine (fg/mL)	Types of treatment			p

	Insulin (n = 18)	Oral antidiabetic drugs (n = 17)	Insulin plus oral antidiabetic drugs (n = 67)	
TNF-α	94 (51)	74 (35)	92 (51)	0.440
IL-6	911 (1546)	729 (877)	783 (1518)	0.530
IL-10	187 (63)	180 (42)	194 (78)	0.511

TNF-α: tumor necrosis factor-alpha; IL-6: interleukin-6; IL-10: interleukin-10.

Cytokine levels: no normal variable; the data are shown as “median (IQR)”.

p < 0.05 was considered statistically significant.

**Table SM2:** Distributions of genotypes and alleles frequencies in T2DM patients and controls

Polymorphisms		T2DM (n = 102) (%)	Control (n = 62) (%)	p
TNF-α (-308 G/A) (rs1800629)	Genotypes			
	GG	78 (76.5)	47 (75.8)	0.965
	GA	23 (22.6)	15 (24.2)	
	AA	1 (0.9)	0	
	Alleles			
	G	179 (87.8)	109 (87.9)	0.966
	A	25 (12.2)	15 (12.1)	
IL-6 (-174 C/G) (rs1800795)	Genotypes			
	CC	7 (6.8)	3 (4.8)	0.348
	CG	26 (25.5)	24 (38.7)	
	GG	69 (67.7)	35 (56.5)	
	Alleles			
	C	40 (19.6)	30 (24.2)	0.326
	G	164 (80.4)	94 (75.8)	
IL-10 (-1082 G/A) (rs1800896)	Genotypes			
	GG	12 (11.8)	8 (12.9)	0.864
	GA	47 (46.1)	26 (41.9)	
	AA	43 (42.1)	28 (45.2)	
	Alleles			
	G	71 (34.8)	42 (33.9)	0.863
	A	133 (65.2)	82 (66.1)	
IL-10 (-819 T/C) (rs1800871)	Genotypes			
	TT	13 (12.8)	7 (11.3)	0.674
	TC	40 (39.2)	29 (46.8)	
	CC	49 (48.0)	26 (41.9)	
	Alleles			
	T	66 (32.4)	43 (34.7)	0.665
	C	138 (67.6)	81 (65.3)	
IL-10 (-592 A/C) (rs1800872)	Genotypes			
	AA	14 (13.7)	7 (11.3)	0.742
	AC	39 (38.3)	29 (46.8)	
	CC	49 (48.0)	26 (41.9)	
	Alleles			
	A	67 (32.8)	43 (34.7)	0.733
	C	137 (67.2)	81 (65.3)	

TNF-α: tumor necrosis factor-alpha; IL-6: interleukin-6; IL-10: interleukin-10.

p < 0.05 was considered statistically significant.
